# Visual Timing of Structured Dance Movements Resembles Auditory Rhythm Perception

**DOI:** 10.1155/2016/1678390

**Published:** 2016-05-30

**Authors:** Yi-Huang Su, Elvira Salazar-López

**Affiliations:** Department of Movement Science, Faculty of Sport and Health Sciences, Technical University of Munich, 80992 Munich, Germany

## Abstract

Temporal mechanisms for processing auditory musical rhythms are well established, in which a perceived beat is beneficial for timing purposes. It is yet unknown whether such beat-based timing would also underlie visual perception of temporally structured, ecological stimuli connected to music: dance. In this study, we investigated whether observers extracted a visual beat when watching dance movements to assist visual timing of these movements. Participants watched silent videos of dance sequences and reproduced the movement duration by mental recall. We found better visual timing for limb movements with regular patterns in the trajectories than without, similar to the beat advantage for auditory rhythms. When movements involved both the arms and the legs, the benefit of a visual beat relied only on the latter. The beat-based advantage persisted despite auditory interferences that were temporally incongruent with the visual beat, arguing for the visual nature of these mechanisms. Our results suggest that visual timing principles for dance parallel their auditory counterparts for music, which may be based on common sensorimotor coupling. These processes likely yield multimodal rhythm representations in the scenario of music and dance.

## 1. Introduction

To appreciate means of communication unique to humans, such as music, speech, or dance, the perceptual system needs to keep track of the dynamic information unfolding over time [1]. Beyond simple interval timing [2], current understanding of more complex temporal processes, such as rhythm and beat perception, is mainly derived from findings of music [3] and speech [4] in the auditory domain. This, however, overlooks the fact that amongst the abundant* visually* available information, human movements (e.g., walking) are also often rhythmic [5], for which there is little knowledge how their temporal structure is visually perceived. In this study, we investigated timing mechanisms employed in visual perception of dance movements, a class of movements most immediately linked to musical rhythms [6]. We aimed to establish whether mechanisms adopted for processing auditory rhythms would be similarly found for ecological visual stimuli.

Timing especially in the range of hundreds of milliseconds forms the basis for rhythm perception. In this range, purely perceptual timing without requiring a motor task implicates cortical motor systems [7], supporting the idea that sensory and motor timing share common mechanisms within the time scale that is relevant for movement execution [8, 9]. In the same range, two modes of auditory timing have been distinguished, each subserved by a different motor circuitry that may work as a unified system [10]: the* duration-based* mechanism, which times the absolute interval duration in a sequence without a perceivable beat, and the* beat-based* mechanism, which relies on a perceived beat in a sequence as reference for timing an interval.

Rhythm perception entails tracking the underlying periodicity, such as a beat or pulse, in a temporal pattern of (often auditory) events [3]. It represents a subset of perceptual timing that especially engages motor activities. The audio-motor link has been shown externally as body movements assisting pulse extraction [11] and event timing [12] in auditory rhythms. Internally, beat perception implicates motor areas of the brain and is modulated by their connection to the auditory area [3]. While beat-based mechanism is not superior to duration-based in timing a single auditory interval [10], the presence of a beat facilitates perception of an auditory rhythm (consisting of successive intervals) as a whole [13, 14]: for example, the patterns of auditory rhythms with a perceivable beat can be more accurately reproduced or recalled than those without a clear beat. One explanation of the beat advantage is that beat-based rhythms effectively couple humans' internal motor system, which in turn enhances rhythm perception [15].

Most studies in timing and rhythm perception converge to show auditory superiority compared to its visual counterpart, which may be attributed to a stronger link to the motor system in the former [16]. However, recent findings point to possible visual rhythm and beat perception in moving stimuli [15, 17], particularly for periodic movements of a biological motion profile [18, 19]. The significance of biological motion in timing is also supported by the literature that there seems to be a specialized timing mode for movements of biological kinematics compared to nonbiological ones [20, 21]. Furthermore, human movement kinematics facilitates temporal prediction of an action, compared to motions of artificial, linear velocity [22, 23], which is consistent with the internal motor simulation account during movement observation [24], as well as embodied theories of temporal processing [25]. As such, questions arise as to whether the sensorimotor coupling underlying rhythmic timing can be strengthened by visual observation of temporally structured biological motion [19] and whether this leads to visual timing behaviors similar to those found for auditory rhythms. One type of human movements, dance, provides suitable visual stimuli for addressing this issue, as dance is often performed in time with musical rhythms and may thus communicate visual spatiotemporal rhythms by observation [26]. Moreover, as dance entails whole-body movements, dance observation may activate internal motor representations [27] more effectively than simple, artificial moving stimuli [15, 17], which can be useful for visual timing purposes.

We designed the present study as a novel investigation of visual timing mechanisms during observation of realistic dance movements, focusing on possible beat-based advantages in this process. As periodic biological motion (whole-body bouncing) has been proposed to serve a visual beat [18, 19], we extended the idea to various movements in three experiments, using a duration reproduction task [28]. This task was chosen for the reason that it has not been established whether and how rhythms are visually perceived when observing realistic human movements. As opposed to various perceptual tasks typically employed to measure auditory rhythmic timing, no visual paradigm involving complex movements has yet been developed for this purpose. We thus probed visual temporal perception of a movement sequence in which the feature in question, a potential beat, was embedded or not. The perceived sequence duration and how well it was encoded would likely reflect the movement information during the sequence [29]. We hypothesized that when observing movements involving one or more body parts, periodic limb trajectories, such as recurrent hand clapping or foot tapping, would serve a visual beat. We expected such a visual beat to afford a beat-based mechanism that would benefit visual timing of the whole movement sequence.

Experiment  1 examined movements of the upper and the lower limbs separately. Participants watched short silent videos of a dancer moving with the arms or with the legs. The movements consisted of periodic trajectories (clapping or stepping), continuous and nonperiodic trajectories (circular movements), or a mixture of both interspersed. Participants memorized each sequence and reproduced the duration by mental recall. We expected that movements with periodic trajectories would be better timed than those without. In Experiment  2, we presented movements performed by both the arms* and* the legs, each of which could contain periodic trajectories or not. We examined whether the arms, the legs, or both yielded the salient beat in visual timing of whole-body movements. In Experiment  3, we verified whether the beat advantage in visual timing was attributed to auditory imagery of the impact sounds, by presenting auditory interferences during the same visual task. If the beat advantage persisted, it would argue for visual beat-based timing that is* not* transformed into auditory representations.

## 2. Experiment 1

We examined whether the arm or the leg movements with periodic trajectories were better timed visually than those without and whether the effect varied across different tempi. For the purpose of cross-modal comparison, a similar auditory timing task was also included, in which an auditory sequence could either contain a beat or not. We expected similar patterns of results for the visual and the auditory tasks: namely, better temporal perception for sequences with a beat than those without, within each modality.

### 2.1. Method

*Participants*. Twenty-two healthy volunteers (eleven male, mean age 27 years, SD = 4) took part in this experiment. Participants in all the experiments in this study were naïve of the purpose, gave written informed consent prior to the experiment, and received an honorarium of 8€ per hour for their participation. Participants were not prescreened for musical or dance training, and the training duration ranged from zero to fifteen years for music and zero to six years for dance. Eight participants had received music training (all amateurs), and the learned instruments included piano/keyboard (4), guitar (3), and flute (1). Five participants had received dance lessons (all amateurs). The study had been approved by the ethic commission of Technical University of Munich and was conducted in accordance with the ethical standards of the 1964 Declaration of Helsinki.

### 2.2. Stimuli and Materials

#### 2.2.1. Visual Stimuli

The visual stimuli consisted of videos of six kinds of movement sequences derived from the Flamenco dance repertoire. Each movement sequence was performed in five different tempi, yielding thirty different videos. The movements were chosen based on the moving body part, the body positioning in space, and the direction of the body motion, following criteria similar to those employed by Calvo-Merino et al. [30]. The rationale of employing specific Flamenco movements was that (1) the chosen movements were not too complex for nondancers to imagine performing, thus minimizing potential effects of different sensorimotor experiences across expertise [31], and (2), at the same time, the specific postures and kinematics performed by a trained dancer, preserving the characteristic movement “accent” (in Flamenco term), distinguished these movements from everyday actions, thus maintaining the ecological plausibility of a dance scenario.

We defined a “movement sequence” here as a set of movements that lasted a certain duration from the start to the end posture. To generate the stimuli, we first choreographed each movement sequence with the knowledge of the second author, who holds a degree in Flamenco dance. The sequences were choreographed based on the planned experimental variables, while keeping the movements as characteristic of Flamenco as possible. We then recorded a professional Flamenco dancer (15 years of training) performing each of the six movement sequences paced by metronomes of five different tempi, corresponding to an interbeat interval (IBI) of 375 ms, 425 ms, 500 ms, 575 ms, and 625 ms (i.e., 500 ms ±0, ±15%, and ±25%). Each complete movement sequence corresponded temporally to eight IBIs at the respective tempo, equaling eight beats in a 4/4 musical meter. The dancer practiced each movement sequence until she could perform it fluently to all the metronome tempi. For each movement at each tempo, we recorded the dancer performing at least four cycles of the sequence continuously, one of which was selected later as the visual stimulus. The recordings were made with a camcorder (Panasonic HC-V500) at 25 frames per second in a dance studio against a white background. A spatial reference of 2.5 m × 1.75 m was marked, creating a 6.5 m^2^ space in which every sequence was performed.

The videos were later edited on a frame basis using the software iMovie (Apple, Inc.). For each movement sequence, we defined a starting posture and an end posture in the video as encompassing a complete movement cycle. We then selected one cycle (corresponding to the eight-beat count) at each tempo that yielded the highest consistency of start and end postures with the same movement at the other tempi, as well as the best match to the duration of eight IBIs. Given the natural variability in real human movements and the fact that the kinematics of the same movement varied slightly when performed in different tempi (or speed), for each sequence we allowed five additional frames (i.e., 200 ms) to the intended eight-beat duration to ensure that each selected movement cycle could be fully and consistently presented across all the tempi. The total duration of a sequence at each tempo, shown as video, was thus 3200 ms, 3600 ms, 4200 ms, 4800 ms, and 5200 ms, respectively. Each sequence was exported as an  .m4v file for playback in Matlab (2012b).

The movements varied according to two variables of interest: the limbs used to perform the movement (arms or legs) and the type of movement (with or without periodic trajectories, or a mixture of both). In all the movements the dancer faced the front. In the arms-only movements, the dancer's legs stood still with the feet separated by around 30 cm (basic Flamenco posture). In the legs-only movements, the dancer placed her hands on each side of the hips so that the arms did not move. Regarding the movement type, movements containing periodic limb trajectories were marked by successive, brief contact points such as handclaps or foot taps. For labeling purpose, we termed this movement type* discrete* to reflect the brief moments of discrete contact. We termed movements that did not contain such recurrent contact points as* continuous*, as the limbs moved continuously in a circular manner. Movements that contained components from both* discrete* and* continuous* types were termed* mixed*. It should be noted that the dancer performed all types of movement paced by the metronome; while it is more self-evident that* discrete* movement trajectories could be temporally segmented by the metronome beats, the dancer applied the same principle of segmentation in* continuous* movements, such that the limbs reached a defined body position at each given beat, regardless of movement tempo. The critical difference between these two movement types thus lied in the recurrent patterns, the absence of which made a movement continuous in our scenario.

In the following sections, we describe each movement sequence with reference to the metronome beat count that was used to pace the dancer's movements. Nomenclature of the Flamenco repertoire was supplemented where necessary. See also Table 1 for an overview of the movement and the limb displacement (total traveled distance) in each sequence. Limb displacements were calculated for sequences at the middle tempo (IBI = 500 ms), which should be most representative of the kinematics of each movement type.

**Figure 1:**
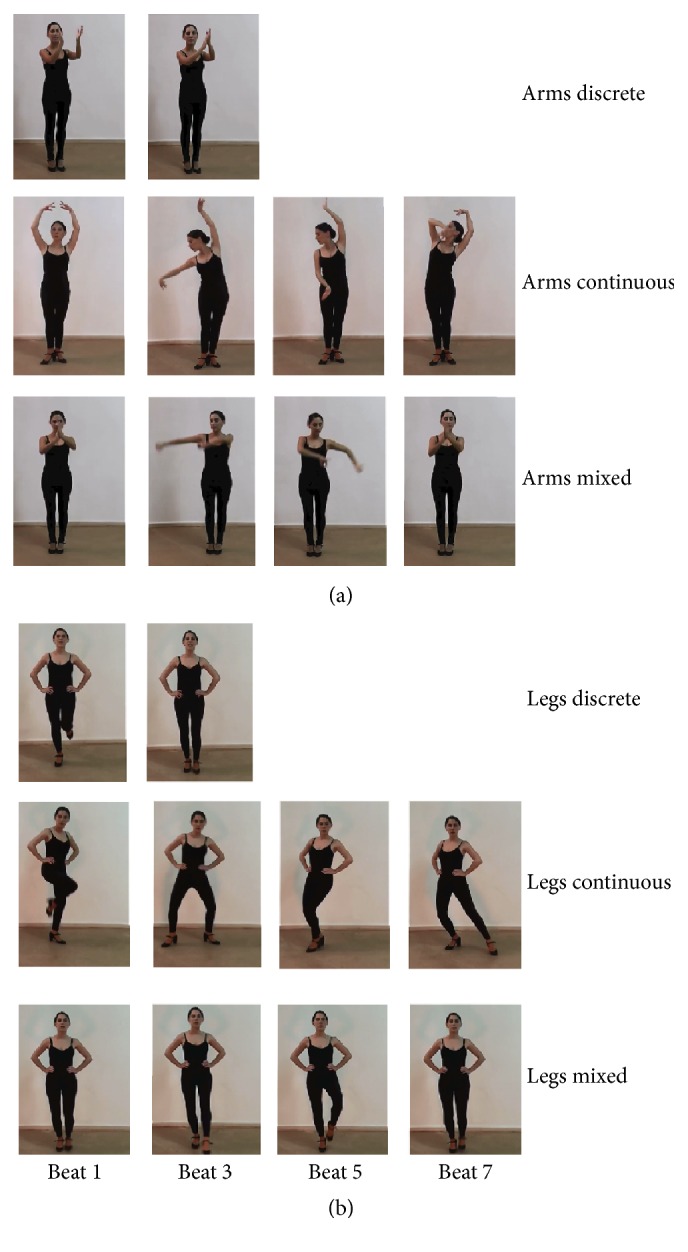
Illustrations of the visual stimuli for Experiment  1, shown as selected frames taken from the videos. (a) 1st row:* discrete* arm movement shown as one handclap. As this trajectory was repeated in the sequence, only one example is shown. 2nd row:* continuous* arm movement. 3rd row:* mixed* arm movement. (b) 1st row:* discrete* leg movement shown as one foot tap. As this trajectory was repeated in the sequence, only one example is shown. 2nd row:* continuous* leg movement. 3rd row:* mixed* leg movement. With the exception of the* discrete* arm and leg movements, the four frames for all the sequences correspond to beats 1, 3, 5, and 7 of the 8-beat count in the metronome that paced the dancer's movement.

*(1) Discrete Movements*. Trajectories were recurrent and gave rise to successive contact points with an underlying periodicity. (1A) The arm movement sequence was based on “Toque de Palmas,” where the dancer held her forearms in front of the face and clapped her hands on the left-frontal side of the body. The dancer started with the two hands in a closed position and clapped six times (on beats 1, 2, and 3 and 5, 6, and 7 of the eight-beat count). See Figure 1(a), 1st row. (1B) The leg movement sequence was derived from “Zapateado,” in which the dancer started with the standing position and made alternating foot taps on the ground (without horizontal translational motion) on beats 1, 2, and 3 and 5, 6, and 7. The taps were made in the following order of the foot: right-right-left, left-left-right (Figure 1(b), 1st row). Note that, in these* discrete* movements, the sequence was defined to start at the beginning of the limb trajectory leading to the first contact point (beat 1), instead of at the first contact point per se.

*(2) Continuous Movements*. Trajectories were nonrecurrent and circular. (2A) The arm movements were derived from “Braceo.” The dancer started with both hands held above the head and moved the right hand downward to the hip level in a circular manner (the arm trajectory similar to that of the arm of a clock) and then upwards in front of the trunk until the two hands were joined above the head in the end. The left hand remained above the head throughout the sequence. The downward and upward movement occupied four beats each. The arm movements were accompanied by hand gestures through wrist rotation that was typical of Flamenco. See Figure 1(a), 2nd row. (2B) In the leg movement, the dancer started with both feet on the ground, lifted the right leg up and down to the ground again to the right side of the body while shifting the hip balance rightward (beats 1 to 4), and then drew a circle on the ground with the left leg in front of the body (beats 5 to 8) that ended by the left foot joining the right (Figure 1(b), 2nd row).

*(3) Mixed Movements*. Segments of* discrete* and* continuous* movements were combined within a sequence. (3A) The arm sequence started with two handclaps in front of the face (“Toque de Palmas,” beats 1 and 2), followed by a continuous trajectory of both arms drawing a circle in parallel in the frontal-coronal plane, stretching above the head and back to the face level (bimanual variation of “Braceo,” beats 3 to 6), and ended with another two claps (beats 7 and 8) in front of the face (Figure 1(a), 3rd row). (3B) The leg sequence started with two taps on the ground by the left foot (beats 1 and 2), followed by the left leg drawing a full circle above the ground in the transverse plan (beats 3 to 6) and back with two more taps on the ground (beats 7 and 8). See Figure 1(b), 3rd row.

#### 2.2.2. Auditory Stimuli

The auditory stimuli consisted of two types of sound sequences, discrete or continuous, each lasting the same five durations as those of the visual stimuli. The* continuous* sequence was a tone lasting one of the five durations, made up of continuously frequency-modulated linear sine sweeps that went from 600 Hz to 200 Hz in the first half of the stimulus duration and from 200 Hz back to 600 Hz in the second half (resembling a siren sound). In the* discrete* sequence, six discrete tones (i.e., six beats) were embedded in the same continuous sequence as described above. The discrete tone was of a synthesized sound of the instrument “clave” with 43 ms tone duration. The beats followed the same temporal structure as the claps or the steps in the visual discrete movement, that is, occupying beats 1, 2, 3, 5, 6, and 7 of an eight-beat count, with an IBI of 375 ms, 425 ms, 500 ms, 575 ms, and 625 ms for the respective sequence duration. The first beat always appeared at 200 ms after the onset of the continuous pitch sweeps. It should be noted that the* discrete* auditory sequence consisted of both a continuous sound and the discrete beats in parallel, the reason for which was to present comparable visual and auditory stimuli: The visual* discrete* movements contained continuously varying spatiotemporal information (i.e., velocity) in the trajectory between successive contact points. We reasoned that this should be more closely mirrored in a continuous sound whose rate of frequency sweeps also scaled according to the sound duration, with discrete beats on top of it, instead of successive beats bordering empty temporal intervals.

### 2.3. Procedure and Design

The experimental program was controlled by a customized Matlab script using Psychophysics Toolbox version 3 [32] routines running on a Mac OSX environment. The visual stimuli were displayed on a 17-inch CRT monitor (Fujitsu X178 P117A) with a frame frequency of 100 Hz at a spatial resolution of 1024 × 768 pixels. The videos were displayed at 960 × 540 pixels. Participants sat with a viewing distance of 80 cm. Sounds were presented at a sampling rate of 44,100 Hz through closed studio headphones (AKG K271 MKII).

Two timing tasks were presented in a blocked manner: a visual task and an auditory task, with the former always preceding the latter. Participants self-initiated each trial when they were ready. In the visual task, participants observed on each trial a short silent video of a dancer performing a movement sequence as described in the visual stimuli. Participants were informed that there were different movement speeds across different trials. We used the term “speed,” instead of “tempo,” as participants more easily understood the former where human movements were concerned. Participants were required to attend to the sequence carefully and to memorize its entire duration. Immediately following the video a reminder text was briefly shown (“Please reproduce the duration now!”), after which an image of the dancer, taken from the first frame of the video, was displayed on the screen. As soon as this image was shown, participants were required to start reproducing the duration by mentally replaying the memorized movement sequence once. They were instructed to do so as closely to the movement speed of the video as possible. Participants indicated the end of duration reproduction by pressing a predefined key once. The image stayed on the screen during their mental recall until key-press.

In the auditory task, participants underwent a similar procedure of duration reproduction with the auditory stimuli as described above. During auditory stimulus presentation and the reproduction phase, only a fixation cross was shown in the middle of the screen, which participants should fixate. For both the visual and auditory tasks, participants were especially instructed not to use any explicit strategies such as counting or moving along [11] but should rather do so by mere observation and listening, respectively. At the end of the entire experiment, each participant was briefly interviewed for any strategies they had adopted to perform each task.

The visual task followed a 2 (limb type) × 3 (movement type) × 5 (tempo) design, each with 10 repetitions (see [28]), and the total trials were presented in five blocks of about 15 minutes each. The auditory task followed a 2 (sound type) × 5 (tempo) design, each with 10 repetitions, presented in five blocks of around 5 minutes each. All the conditions were presented in a balanced manner across blocks, with the order of conditions randomized within a block. Participants underwent five practice trials prior to the visual and the auditory task, respectively. Every participant completed the visual blocks before starting the auditory ones, as we intended to avoid introducing the idea of auditory imagery for the visual task. The entire experiment was completed in about two hours, and a break was required after each block.

### 2.4. Analyses

No participant reported substantial difficulty in carrying out the tasks. In the rare occasions where a response was given by mistake before the duration reproduction was carried out (if a reproduced duration was shorter than 1500 ms, which exceeded three standard deviations from each within-participant mean), the trial was considered as errors and discarded from analyses. This constituted on average only 0.76% of the trials.

Three parameters were analyzed individually for each condition to index the performance of duration reproduction [33]: (1) Absolute Error (AE), calculated as the absolute deviation of the reproduced interval from the presented one, in percentage. A greater AE indicates a larger error in duration reproduction. (2) Ratio, calculated as the reproduced duration divided by the presented duration. A ratio of one signifies perfect reproduction, and a ratio smaller/larger than one represents underestimation/overestimation of the duration. (3) Coefficient of Variation (CV), calculated for a given condition as the within-participant standard deviation of the reproduced intervals divided by his/her mean reproduced interval, shown in percentage [34]. CV indexes the consistency of duration perception and reproduction; a greater CV signifies more variable reproduction and thus poorer performance. As the present task required timing the durations of movement sequences with varying embedded temporal structures, the perceptual mechanism was expected to resemble that for timing the pattern of an auditory rhythm (as opposed to timing a duration without content) [29]. While AE and ratio indexed how accurately a sequence was estimated in absolute terms, there could be systematic over- or underestimation due, for example, to Vierordt's law across sequence tempi [35, 36], or due to individual differences in the tendency to over- or underreproduce [13, 37], which is not necessarily associated with the presence or absence of a beat. In comparison, timing variability as indexed by CV may be more immune to these factors and able to reflect the rhythmicity of the movement [38]. As such, along with AE and ratio that describe timing behaviors, CV would be taken as the more indicative measurement of the present task.

Data from one participant were excluded from further analyses, as the intervals were overall substantially underreproduced (mean ratio = 0.63 and mean AE = 37%, which was the only case from the whole sample exceeding two standard deviations of the sample mean in both parameters). This suggests that the participant either did not fully understand the task or was hurrying through each trial without proper recall of the stimulus. The sample size for the reported results was therefore 21.

For all the repeated-measures ANOVAs and ANCOVAs reported in this study, Greenhouse-Geisser correction was applied to the *p* values of effects of variables with more than two levels. Tukey HSD was used as post hoc tests following a significant main effect.

### 2.5. Results

#### 2.5.1. Visual Task

First we provide an overview of the strategies participants (*N* = 21) reported of adopting for the visual task: fourteen participants reported associating sounds along with visual imagery to aid mental replay, eight of whom used the auditory strategy only for the* discrete* movements (i.e., as if they could hear the impact sounds in their head). The others used only visual imagery for the visual task.

For each of the three parameters, we conducted a 2 (limb type) × 3 (movement type) × 5 (tempo) repeated-measures ANCOVA of the individual means, with individual music or dance training duration entered as covariate in each analysis. We pulled together training in music and dance as one general category of rhythm-related expertise that may influence performance in the present task.

**Figure 2:**
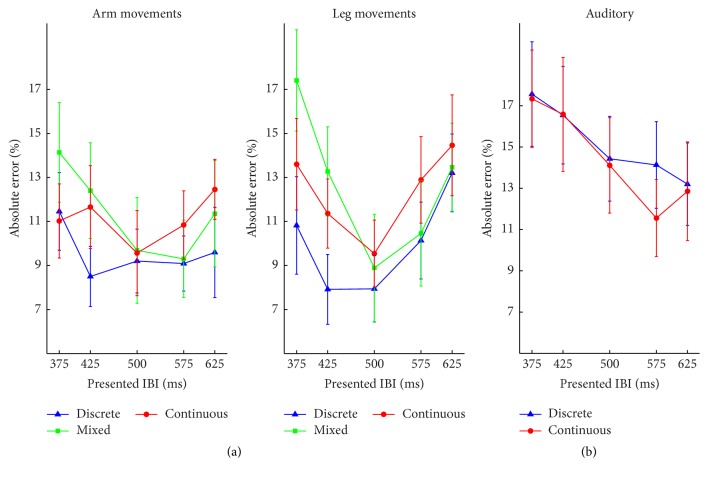
Mean Absolute Error (AE) of Experiment  1, for each experimental condition as a function of the movement tempo. Tempo is labeled as the metronome IBI (in ms) used to pace the dancer's movements. (a) Results of the visual task. (b) Results of the auditory task. Error bars represent standard error of the means.

*AE*. Only a significant main effect of movement type was found, *F*(2, 38) = 3.95, *p* = 0.028, and *η*_*p*_^2^ = 0.20, and the post hoc tests showed that AE was lower for* discrete* than for either* mixed*, *p* = 0.02, or (almost)* continuous*, *p* = 0.058, while the latter two did not differ from each other. The interaction between limb type and tempo was significant, *F*(4, 76) = 3.73, *p* = 0.025, and *η*_*p*_^2^ = 0.16, which was also modulated by the covariate of training duration, *F*(4, 76) = 3.29, *p* = 0.038, and *η*_*p*_^2^ = 0.15. Following this interaction, post hoc comparisons (Bonferroni corrected) for the arm movements did not identify any difference amongst different tempi, all *p*s > 0.5, while for the leg movements AE in the middle tempo (IBI = 500 ms) was lower than that in the two slowest tempi (IBI = 575 and 625 ms), *p* = 0.028 and *p* = 0.014, respectively. To examine how expertise modulated this effect, Pearson's correlations (*N* = 21) were computed between training duration and AE of leg movement for the three slower tempi, which revealed a significant negative correlation between AE and training duration at the slowest tempo (IBI = 625 ms), *r* = −0.44 and *p* = 0.04, and marginally so at the next slowest (IBI = 575 ms), *r* = −0.41 and *p* = 0.06. No other significant effects were found: limb type, *F*(1, 19) = 1.60, *p* = 0.22, and *η*_*p*_^2^ = 0.078, and tempo, *F*(4, 76) = 1.06, *p* = 0.34, and *η*_*p*_^2^ = 0.053 (Figure 2(a)). Training duration did not interact with any other effects, all *p*s > 0.2 and all *η*_*p*_^2^ < 0.08.

*Ratio*. First, a main effect of tempo was found, *F*(4, 76) = 39.73, *p* < 0.001, and *η*_*p*_^2^ = 0.68, with the post hoc tests showing that the reproduced ratio for the two fastest tempi was greater than that for the two slowest ones, all *p*s < 0.001. The reproduced ratio for the middle tempo (IBI = 500 ms) also differed from those for the two fastest ones, both *p*s < 0.002, as well as from those for the two slowest ones, both *p*s < 0.05. On average, participants' reproduced ratio descended across decreasing tempo, with overestimation for the faster ones and underestimation for the slower ones. Main effects of limb type and movement type were not significant, *F*(1, 19) = 2.81, *p* = 0.11, and *η*_*p*_^2^ = 0.13 and *F*(2, 38) = 0.91, *p* = 0.40 and *η*_*p*_^2^ = 0.046.

**Figure 3:**
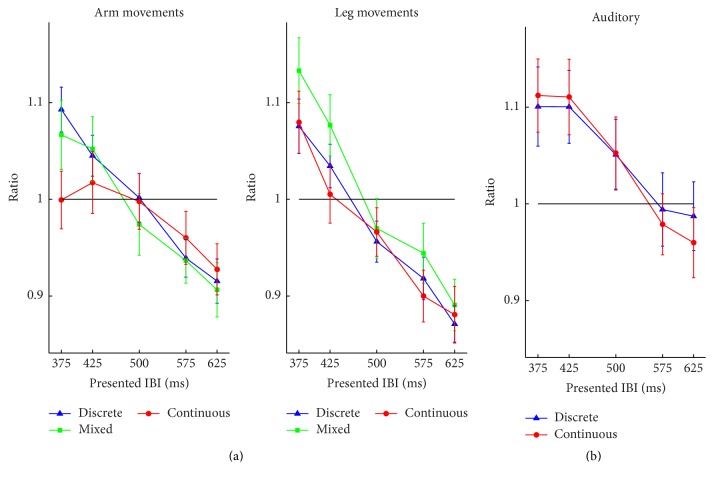
Mean ratio of Experiment  1, for each experimental condition as a function of the movement tempo. (a) Results of the visual task. (b) Results of the auditory task. Error bars represent standard error of the means. The black horizontal lines depict a ratio of 1, which would be perfect reproduction.

Following a significant three-way interaction, *F*(8, 152) = 4.42, *p* = 0.002, and *η*_*p*_^2^ = 0.19, follow-up two-way ANOVAs were conducted for each limb type separately. For the arm movements, the movement type × tempo interaction was significant, *F*(8, 160) = 5.95, *p* < 0.001, and *η*_*p*_^2^ = 0.23, and the post hoc one-way ANOVAs for each tempo separately showed that only at the fastest tempo was the ratio different between* discrete* and* continuous* movements, *p* = 0.008 (Bonferroni corrected), while no effect of movement type was found in all the other tempi. For the leg movements, a main effect of movement type was found, *F*(2, 40) = 3.61, *p* = 0.04, and *η*_*p*_^2^ = 0.15, the post hoc test showing a trend of greater reproduced ratio for* mixed* than for* continuous* movements, *p* = 0.08. The movement type × tempo interaction was only marginally significant, *F*(8, 160) = 2.32, *p* = 0.054, and *η*_*p*_^2^ = 0.10 (Figure 3(a)). Training duration as covariate did not interact with any of the effects, all *p*s > 0.15 and all *η*_*p*_^2^ < 0.08.

**Figure 4:**
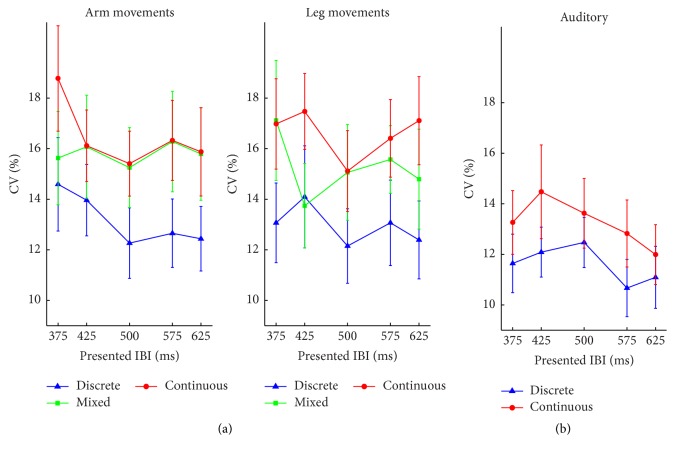
Mean Coefficient of Variation (CV) of Experiment  1, for each experimental condition as a function of the movement tempo. (a) Results of the visual task. (b) Results of the auditory task. Error bars represent standard error of the means.

*CV*. The main effect of movement type was significant, *F*(2, 38) = 17.40, *p* < 0.001, and *η*_*p*_^2^ = 0.48, with post hoc tests showing a lower CV for* discrete* than for either* continuous* or* mixed* movements, both *p*s < 0.005, while the latter two did not differ from each other. The main effect of tempo was marginally significant, *F*(4, 76) = 2.66, *p* = 0.055, and *η*_*p*_^2^ = 0.12. There was no effect of limb type, *F*(1, 19) = 0.014, *p* = 0.908, and *η*_*p*_^2^ = 0.001 (Figure 4(a)). Training duration as covariate did not interact with any of the effects, all *p*s > 0.16 and all *η*_*p*_^2^ < 0.04.

#### 2.5.2. Auditory Task

For the auditory task, five participants reported visualizing the sounds, and two of them did so especially for continuous auditory sequences. The majority of the participants adopted only auditory imagery. Individual means of each of the three parameters were submitted to a 2 (sound type) × 5 (tempo) repeated-measures ANCOVA, with training duration as covariate.

*AE*. The analysis did not reveal any significant effect of the variables, sound type, *F*(1, 19) = 0.01, *p* = 0.92, and *η*_*p*_^2^ = 0.001, and tempo, *F*(4, 76) = 1.36, *p* = 0.26, and *η*_*p*_^2^ = 0.067, or interaction, *F*(4, 76) = 1.34, *p* = 0.27, and *η*_*p*_^2^ = 0.066. (Figure 2(b)). Training duration did not interact with any variable, all *p*s > 0.2 and all *η*_*p*_^2^ < 0.1.

*Ratio*. Only a significant main effect of tempo was found, *F*(4, 76) = 21.09, *p* < 0.001, and *η*_*p*_^2^ = 0.53. Post hoc comparisons showed that the reproduced ratio for the two fastest sequences was greater than that for the two slowest ones, all *p*s < 0.02 (Figure 3(b)). Effect of sound type was not significant, *F*(1, 19) = 0.8, *p* = 0.38, and *η*_*p*_^2^ = 0.04, nor was its interaction with tempo, *F*(4, 76) = 2.07, *p* = 0.12, and *η*_*p*_^2^ = 0.098. Training duration did not interact with any variable, all *p*s > 0.2 and all *η*_*p*_^2^ < 0.1. On average, as found in the visual task, shorter durations were overestimated while longer ones were underestimated, although the extent of underestimation appeared smaller than in the visual task.

*CV*. The effect of sound type was not significant in the ANCOVA analysis, *F*(1, 19) = 1.76, *p* = 0.2, and *η*_*p*_^2^ = 0.09, though it was in the ANOVA without covariate, *F*(1, 20) = 6.0, *p* = 0.02, and *η*_*p*_^2^ = 0.23 (the covariate did not interact with sound type, *F*(1, 19) = 0.66, *p* = 0.43, and *η*_*p*_^2^ = 0.03). No other effect was significant: tempo, *F*(4, 76) = 1.75, *p* = 0.16, and *η*_*p*_^2^ = 0.084, and interaction, *F*(4, 76) = 0.71, *p* = 0.53, and *η*_*p*_^2^ = 0.036. Training duration only interacted with tempo, *F*(4, 76) = 2.84, *p* = 0.04, and *η*_*p*_^2^ = 0.13 (Figure 4(b)).

 In sum, results of the visual task showed that, regardless of the limbs performing the movements and the movement tempo,* discrete* movements led to more accurate (lower AE) and more consistent (lower CV) temporal reproduction than both the* continuous* and the* mixed* movements, while performance in the latter two did not differ from each other. Timing for the leg movements was more accurate (lower AE) in the middle tempo compared to the two slowest tempi; this effect was modulated by expertise, such that longer training duration was associated with lower AE in the two slowest tempi. Besides, faster movements tended to be overestimated and slower ones underestimated. For the auditory task, the pattern of ratio was similar to that in the visual task, with overestimation and underestimation for the faster and slower tempi. The effect of a discrete beat on auditory timing was not robust enough to survive the analysis with the covariate included.

## 3. Experiment 2

Following results of Experiment  1, we examined whether the arms, the legs, or both, in a multi-limb movement sequence accounted for the beat advantage in visual timing. Applying the same visual timing paradigm, we presented now movements performed by both the arms and the legs, each of which could be either discrete or continuous.

### 3.1. Method

*Participants*. Twenty healthy volunteers (seven male, mean age 25 years, SD = 3) took part. Thirteen participants had received music training ranging from three to seventeen years (all amateurs), and the instruments included piano/keyboard (6), guitar (4), trumpet (1), oboe (1), and cello (1). Three participants had received dance lessons between one and three years. Seven of the participants had participated in Experiment  1 two to four weeks earlier.

### 3.2. Stimuli and Materials

Only visual stimuli were presented in this experiment, and they consisted of videos of four kinds of movement sequences derived from the Flamenco repertoire. The sequences were performed by the same Flamenco dancer across the same five tempi as in Experiment  1. The same procedures of movement recording and video editing and formatting were applied, yielding the same five sequence durations. All the movement sequences here were performed with the arms* and* the legs. The sequences now varied according to two variables: the arm movement type (discrete or continuous) and the leg movement type (discrete or continuous). See also Table 2 for an overview.

#### 3.2.1. Arms Discrete + Legs Discrete

**Figure 5:**
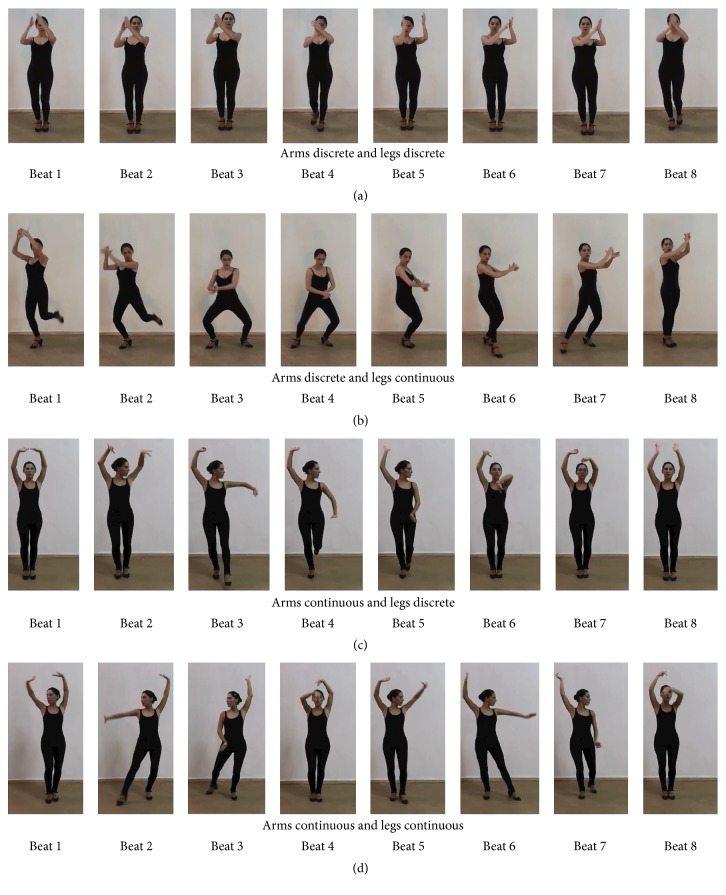
Illustrations of the visual stimuli for Experiment  2, shown as selected frames taken from the videos. (a) Arms discrete and legs discrete. (b) Arms discrete and legs continuous. (c) Arms continuous and legs discrete. (d) Arms continuous and legs continuous. The eight frames for each sequence correspond to the 8 beats in the metronome that paced the dancer's movements.

The dancer made one tap on the ground with the left foot (beat 1), followed by three claps on the right-frontal side of the body (“Toque de Palmas,” on beats 2, 3, and 4), and then another tap with the right foot (beat 5), followed by three more claps on the left-frontal side of the body (beats 6, 7, and 8). The start of the sequence followed the same rule as previously described for the* discrete* movements. See Figure 5(a).

#### 3.2.2. Arms Discrete + Legs Continuous

Discrete claps were combined with the continuous leg movement as described in Experiment  1. The dancer held her arms at the head level and clapped three times along the right-frontal plane of the body (beats 1, 2, and 3), during which the left leg was lifted and stretched above the ground and down on the left side (beats 1 to 4), followed by another three claps on the left side of the body (beats 5, 6, and 7), during which the right leg drew a circle on the ground in front of the body (beats 5 to 8) that ended by joining where the left foot was. See Figure 5(b).

#### 3.2.3. Arms Continuous + Legs Discrete

The arm movement was similar to the* continuous* one in Experiment  1 (“Braceo”), where the left arm moved downward (beats 1 to 4) and upward again (beats 5 to 8) in a circular manner to eventually join the right arm that was held above the head throughout. In parallel, the legs carried out discrete taps (without horizontal translational motion) derived from the movement “Marcaje,” in which the first right tap (beat 1) was followed by the left foot doing a front kick by sliding the shoe forward (beat 2), one back kick by sliding the shoe backwards (beat 3), and then one down kick by tapping the ground with the toe cap (beat 4) and concluded by three successive left-right-left taps (beats 5 to 7). See Figure 5(c).

#### 3.2.4. Arms Continuous + Legs Continuous

This movement combined similar continuous movements of the arms and the legs as in Experiment 1. The right arm carried out the circular movement (“Braceo”) while the right leg drew a circle on the ground (beats 1 to 4), followed by the same movement pattern performed with the left arm and left leg (beats 5 to 8). See Figure 5(d).

### 3.3. Procedure and Design

The setup was the same as in Experiment  1. Participants performed the visual timing task following the same instruction and procedures as for the previous experiment, with special emphasis on observing the multi-limb movement as a whole instead of focusing on any specific body part. The experiment followed a 2 (arm movement type) × 2 (leg movement type) × 5 (tempo) design, each with 10 repetitions. The total trials were presented in five blocks of about 10 minutes each, with all the conditions presented in a balanced manner across blocks and the order of conditions randomized within a block. The whole experiment was completed within one hour, with a short break after each block.

### 3.4. Results

Erroneous trials with too short intervals (same criterion as in Experiment  1) were discarded, which constituted on average only 0.6% of the trials. Eight participants reported imagining the sounds along with visual imagery for the task, four of whom did so only when there were discrete movements. The majority of the participants reported adopting only a visual imagery strategy. The same three parameters as described in Experiment  1 were analyzed individually, and the individual means of each parameter were submitted to a 2 (arm movement type) × 2 (leg movement type) × 5 (tempo) repeated-measures ANCOVA, with training duration as covariate.

**Figure 6:**
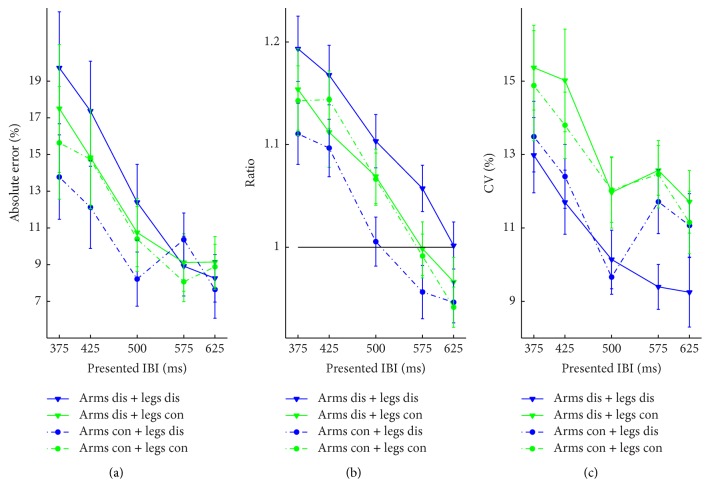
Results of Experiment  2, plotted for each experimental condition as a function of the movement tempo. (a) Mean AE. (b) Mean ratio. (c) Mean CV. Error bars represent standard error of the means.* dis*: discrete;* con*: continuous.

*AE*. No significant main effects or interactions were found, except for the marginally significant effect of arm movement, *F*(1, 18) = 21.09, *p* = 0.077, and *η*_*p*_^2^ = 0.16, and the marginally significant three-way interaction, *F*(4, 72) = 2.76, *p* = 0.051, and *η*_*p*_^2^ = 0.13. Training did not interact with any of the variables (Figure 6(a)).

*Ratio*. The main effect of arm movement was significant, *F*(1, 18) = 19.22, *p* < 0.001, and *η*_*p*_^2^ = 0.52, showing a greater ratio in discrete than in continuous arm movements, but that of leg movement was not, *F*(1, 18) = 0.024, *p* = 0.88, and *η*_*p*_^2^ = 0.001. The main effect of tempo was also significant, *F*(4, 72) = 35.56, *p* < 0.001, and *η*_*p*_^2^ = 0.66, and the post hoc tests showed that while the ratio did not differ between the two fastest tempi or between the two slowest tempi, the two groups differed from each other, as well as from the ratio in the middle tempo, all *p*s < 0.02. As found in Experiment  1, sequences of the faster tempi were on average more overestimated than those of the slower tempi (Figure 6(b)). The arm × leg interaction was marginally significant, *F*(1, 18) = 3.31, *p* = 0.086, and *η*_*p*_^2^ = 0.15. Training duration did not interact with any variable.

*CV*. There was a main effect of leg movement type, *F*(1, 18) = 5.83, *p* = 0.027, and *η*_*p*_^2^ = 0.25, showing lower CV for discrete than for continuous leg movements, but not of arm movement type, *F*(1, 18) = 1.16, *p* = 0.69, and *η*_*p*_^2^ = 0.009. The main effect of tempo was also significant, *F*(4, 72) = 8.59, *p* < 0.001, and *η*_*p*_^2^ = 0.32; post hoc tests showed that CV for the two fastest tempi was higher than that for the other three slower ones, all *p*s < 0.01 (except for *p* = 0.08 between IBIs of 425 ms and 575 ms). No interaction was significant, all *p*s > 0.2 and all *η*_*p*_^2^ < 0.1 (Figure 6(c)). Training duration as covariate did not interact with any of the variables, all *p*s > 0.2 and all *η*_*p*_^2^ < 0.1. 

To summarize, regardless of the movement tempo, discrete leg movements led to more consistent timing (lower CV) than continuous leg movements, while arm movements did not influence CV. Besides, movement tempo affected CV, which was not found in Experiment  1, such that faster movements led to lower consistency in timing than slower ones.

## 4. Experiment 3

The first two experiments showed better visual timing for (especially leg) periodic trajectories marked by discrete contact points, possibly due to a sense of visual beat arising from observing these movements. Here we verified whether this effect was attributed to internalized impact sounds, namely, whether the hypothesized visual beat was obligatorily encoded as auditory representation [39]. We presented the* discrete* and the* continuous* leg movements either in silence, or with task-irrelevant auditory sequences that were temporally congruent or incongruent with the foot taps. If the beat had been encoded auditorily, incongruent interferences would have eliminated the timing advantage of* discrete* movements. If the beat percept remained visual, then the result pattern should persist despite auditory interferences. We included both congruent and incongruent sounds so that, should an auditory interference effect be observed, it could be determined whether it was caused by the temporal structure or the mere presence of the sounds.

### 4.1. Method

*Participants*. Twenty healthy volunteers (nine female, mean age 28 years, SD = 4.6) took part in this experiment, whose musical training duration ranged from zero to twenty years (mean duration 4.9 years, SD = 5). Thirteen participants were musically trained (all amateurs), and the instruments included piano/keyboard (6), guitar (5), trumpet (1), and saxophone (1). No participant in this experiment had received formal dance lessons. Six and four participants had participated in Experiments  1 and 2, respectively, amongst whom two had participated in both.

### 4.2. Stimuli and Materials

#### 4.2.1. Visual Stimuli

The visual stimuli here consisted of videos of two leg movement types as employed in Experiment  1:* discrete* and* continuous*. Two from the five previously displayed tempi, corresponding to an IBI of 425 ms and 575 ms (i.e., the second fastest and the second slowest), were used here.

#### 4.2.2. Auditory Stimuli

The auditory interference in this task consisted of discrete tones of the same clave sound as used in Experiment  1. Two kinds of auditory sequences were presented that were temporally congruent or incongruent with the timing of the discrete leg movement. The* congruent* sequence consisted of four discrete tones, which, when presented concurrently to the discrete leg movement, would temporally coincide with four of the six foot taps (see the description of the* discrete* leg movement in Experiment  1). The four positions were randomly selected on a trial basis. The* incongruent* sequence was initially constructed in the same way as the* congruent* one, but each tone was then advanced or delayed for a magnitude of 20% to 40% of the respective IBI. Whether a tone was delayed or advanced, as well as the magnitude of this shift, was determined randomly for each tone on each trial.

### 4.3. Procedure and Design

The setup was the same, and participants performed the visual timing task following the same procedures as described before. In one-third of the trials, videos were presented in silence. In the other two-thirds, sounds were displayed through headphones during the video; half of them were the* congruent* sequences, and the other half were the* incongruent* ones. Participants received the same instruction as in Experiment  1 and were additionally informed that they would sometimes hear sounds during the video, which were task irrelevant and should be ignored.

The experiment followed a 2 (leg movement type) × 2 (tempo) × 3 (auditory interference) design, each condition with 10 repetitions. The total trials were presented in three blocks of around 10 minutes each. The whole experiment was completed in about half an hour, with a short break after each block.

### 4.4. Results

Erroneous trials were discarded in which a response was accidentally given too quickly (same criterion as before), which occurred rarely (0.5% of the trials on average). Most participants reported having difficulty ignoring the sounds completely, despite the intention to comply with the instruction. As before, AE, ratio, and CV were analyzed individually and submitted to a 2 (movement type) × 2 (tempo) × 3 (auditory interference) repeated-measures ANCOVA, with training duration entered as covariate.

**Figure 7:**
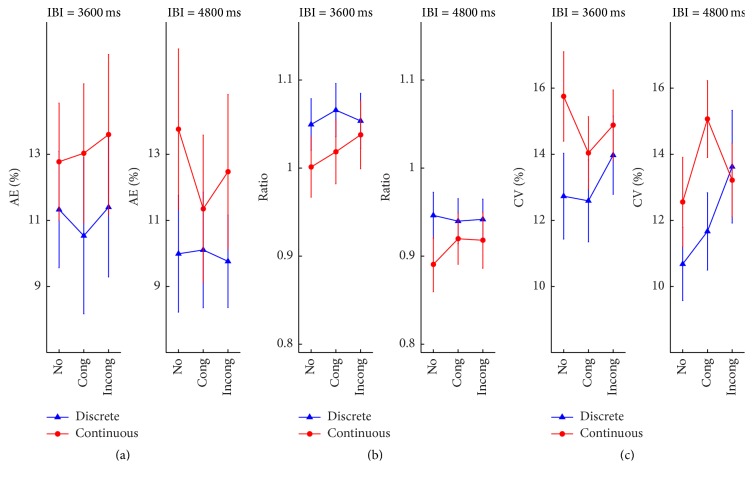
Results of Experiment  3, plotted for each movement type as a function of the auditory interference condition, for each tempo separately. (a) Mean AE. (b) Mean ratio. (c) Mean CV. Error bars represent standard error of the means. On the *x*-axis,* No*,* Cong*, and* Incong* represent no sound, congruent auditory sequence, and incongruent auditory sequence, respectively.

*AE*. No significant effect of any factor was found, movement type, *F*(1, 18) = 1.92, *p* = 0.18, and *η*_*p*_^2^ = 0.097; tempo, *F*(1, 18) = 0.15, *p* = 0.70, and *η*_*p*_^2^ = 0.008; auditory interference, *F*(2, 36) = 0.63, *p* = 0.52, and *η*_*p*_^2^ = 0.034, or any significant interaction (Figure 7(a)). Training duration did not interact with any variable.

*Ratio*. A significant effect of tempo was shown, *F*(1, 18) = 39.86, *p* < 0.001, and *η*_*p*_^2^ = 0.69. Similar to what was previously found, sequences of the faster tempo were overestimated (mean ratio > 1) while those of the slower tempo were underestimated (mean ratio < 1). Although there was a main effect of auditory interference, *F*(2, 36) = 3.61, *p* = 0.041, and *η*_*p*_^2^ = 0.17, post hoc comparisons did not identify any significant difference between conditions, all *p*s > 0.15 (Figure 7(b)). No other effects nor interactions were found significant, and training duration did not interact with any variable.

*CV*. There was a significant main effect of movement type, *F*(1, 18) = 9.95, *p* = 0.005, and *η*_*p*_^2^ = 0.36, showing a lower CV for discrete than for continuous movements. The effect of tempo was only marginally significant, *F*(1, 18) = 3.48, *p* = 0.078, and *η*_*p*_^2^ = 0.16, with a trend of higher CV for the faster tempo. The effect of auditory interference was again not significant, *F*(2, 36) = 1.32, *p* = 0.28, and *η*_*p*_^2^ = 0.068 (Figure 7(c)). No significant interaction was found, nor did training duration interact with any variable.

Compared to when the visual task was performed in silence, the presence of an auditory interference sequence, regardless of its temporal structure, had no influence on any of the measured parameters. The result of more consistent timing in discrete than in continuous movements, as found in Experiment 1, persisted despite the auditory interferences.

## 5. Discussion

We investigated whether perceptual mechanisms similar to those previously found for auditory rhythms, such as beat-based strategies [3], were employed when observing temporally structured dance movements. In all three experiments, we found that periodic limb trajectories benefitted visual timing of a movement sequence, which was most consistently reflected in timing variability (CV). When both the arms and the legs moved, only periodicities in the leg movement accounted for the timing advantage. This advantage persisted despite auditory interferences, suggesting that it was* not* attributed to internal representation of the impact sounds.

We interpret the main result as evidence that observers extracted a visual beat from periodic trajectories [18], which facilitated temporal perception of the whole movement sequence. Notably, the periodic trajectories (handclaps or foot taps) did not necessarily occur on every beat. Their temporal structure resembled non-isochronous auditory rhythms that communicated an underlying beat [13, 40]. Our visual results are thus reminiscent of previous auditory findings that a* perceived* beat leads listeners to adopt a beat-based timing strategy [10] that enhances rhythm perception [13, 14], suggesting similarities between auditory and visual rhythmic timing. The lack of a robust beat effect on improving auditory timing in Experiment  1 might be due to several factors: For one, the auditory stimuli were not as rich and ecological as the visual ones. For another, in terms of contrasting conditions with and without a beat, the auditory stimuli might not have been optimally comparable to their visual counterpart. Perhaps a closer resemblance to the visual discrete condition would have been, for example, successive (shorter) filled intervals yielding the same beat structure. The auditory beat effect might also have been attenuated by the learning effect, as the auditory task was always performed after the visual one. Finally, whereas a picture of the dancer was presented to trigger participants' recall in the visual task, no such rich cues were given prior to the auditory recall, which might have compromised the auditory performance. Thus, different factors deserve consideration when comparing timing behaviors between dance movements and auditory rhythms: naturalistic content [41] or biological motion [18, 21, 23] of visual stimuli may enhance beat advantage in real dance movements, compared to artificial sounds simulating the temporal structure of these movements. In addition, the compatibility of the visual and auditory stimuli yielding the same temporal structure appears critical and needs further verifications.

The beat effect on timing was not modulated by music or dance expertise, suggesting the generality of this mechanism [17]. While it seems fitting to explain our visual results borrowing the framework of auditory timing [10], with beat-based mechanism for* discrete* movements and duration-based mechanism for* continuous* ones, given the differences in paradigms and stimuli, we do not imply that these auditory mechanisms can be directly mapped onto visual timing of realistic human movements. Whether these timing modes are indeed supramodal still warrants further investigations [2, 42]. Similarly, on the basis of shared perceptual and motor timing processes, our perceptual results (for* discrete* versus* continuous* movements) seem reminiscent of the dualistic motor timing in synchronization tasks: discrete movements (e.g., finger-tapping) employ event-based timing, whereas continuous movements (e.g., circle drawing) employ emergent timing [43]. The former carry motor timing advantages over the latter due to their perceivable discrete events (tap contact). It may be tentatively argued that the present timing advantages for beat-based movements arise from perceptual processes corresponding to, or even shared with, their motor counterparts. Furthermore, it has been proposed that these two motor timing modes cannot be combined [44], which seems consistent with our result that adding beat-based components to a non-beat-based movement (*mixed*) did not improve visual timing. Timing difficulty in this case likely arose from the continuous trajectory, which deterred the perceptual system from adopting a beat-based strategy.

One question may arise as to whether the observed advantage of a visual beat in timing was associated with possible counting strategies [45] for* discrete* movements. This explanation was, however, not supported by the result that* mixed* movements, despite the presence of regular trajectories and thus the possibility of counting, were not better timed than* continuous* ones. In addition, counting or segmenting would also have been possible in a* continuous* movement based on positional cues [18] and could thus not exclusively account for improved timing for* discrete* ones. Similarly, one might discuss whether visual timing could have been influenced by stimulus factors such as total traveled distance of the limbs. As shown in Experiment 1 (Table 1), while differences in limb displacement were admittedly hard to control for in real human movements, there was no systematic difference across different movement types or limbs that would correspond to the obtained results (e.g., more consistent timing for* discrete* movements was not associated with more or less limb displacement across limb types). Thus, performance in the present tasks was unlikely to be modulated by such stimulus features.

Our findings also reveal how different parts of a whole-body movement are timed in parallel. While a beat in either the arm or the leg movement assisted visual timing, in a multi-limb movement the beat-based benefit relied only on the legs. It would seem as if observers first oriented to the leg movement for a beat which, if found, enabled them to adopt beat-based timing. If not, however, observers did* not* resort to the arm movement either, even if a beat was available. This pattern suggests that temporal perception of multi-limb movements is somewhat different than can be explained by timing the upper or lower limbs alone, and a higher weight in timing is given to the lower limbs. The fact that the beat-based mechanism is driven by the leg movements seems to fit the action-perception coupling often proposed in rhythm perception: for example, preferred musical tempo corresponds to preferred frequency of locomotion, which concerns mainly the leg movements [5]. Thus, visual timing of dance movements may engage a common sensory-motor platform as for processing auditory rhythms, arguing for the multimodal nature of rhythm representations. It should be noted that the “leg dominance” in visual timing cannot be explained by a preference for the lower visual field alone, as such a preference has mainly been established in goal-directed actions involving tools, and only when viewers are actively engaged in object manipulation, not during passive viewing [46]. In addition, an upper visual field preference has also been found in a visual search task [47]. Thus, a general spatial bias regardless of the visual information does not seem to underlie our finding.

Contrary to earlier proposals that the temporal structures of simpler visual stimuli were obligatorily represented in auditory terms [16, 39], where task-irrelevant sounds were shown to impair visual timing, the present lack of auditory interference effect argues for the visual nature of beat-based timing, at least for rich, ecological movement information, an idea that has received increasing support [19, 48]. The fact that also the congruent sounds had no effect on visual task performance suggests that either the auditory and visual streams were not integrated temporally, or the integration provided no additional assistance to the present task, as the sounds did not offer more beat-related information than the visual stimuli. It would be interesting for future studies to examine whether (task irrelevant) congruent and incongruent visual interferences would influence visual timing in this case. As several participants reported auditory imagery during the visual tasks, we cannot rule out possible auditory co-representations of visual movement rhythms. Although these co-representations may exist in parallel to the visual ones, they did not seem to replace the latter nor influence visual timing. In fact, when movements became more complex (as in Experiment  2), fewer participants reported using auditory strategies, indicating greater reliance on the visual representation. To what extent movement observation elicits auditory co-representations, how the tendency varies with movement complexity, and whether the two sensory representations interact remain interesting questions for follow-up research.

Movement tempo modulated visual timing of whole-body movements in Experiment  2, where slower movements were more consistently timed. As dance observation activates an internal motor program in the observers [27], greater difficulty in simulating these movements at faster tempi may increase difficulty in representing their temporal structures. This interpretation is supported by the fact that movement tempo did not affect visual timing consistency of simpler movements in Experiment  1, which could likely be simulated with equal ease across tempi. There might be a range of optimal tempi for each movement both in execution and in perception, such that movements considerably slower or faster than these tempi are less well represented and thus more difficult to time visually. Movement tempo did, however, influence absolute timing accuracy (AE) of the leg movements in Experiment  1, with more deviation in the two slowest tempi than in the middle one, whereby those with longer music or dance training were less subject to such errors. Thus, while beat-specific effects in visual timing were independent of expertise, training appeared to be beneficial for more general timing functions irrespective of beat, such as absolute duration estimation, in slower movements. Finally, the effect of tempo on ratio observed in both Experiments  1 and 2, namely, more over- and underestimation for faster and slower sequences, respectively, can be explained by Vierordt's law. The fact that shorter and longer intervals tend to be over- and underestimated when presented in the same experiment has been repeatedly reported in the timing literature, which also applies to tempo in a rhythmic context [35, 36, 49].

In conclusion, we presented evidence of visual timing mechanisms for dancelike movements, showing a beat-based advantage that relies especially on the leg trajectories. While they appear similar to mechanisms of auditory rhythm perception found in previous studies, we demonstrated the visual nature of movement timing. These results have implications in how we approach multisensory rhythms in an ecological scenario, which may lead to new research linking action perception and rhythm perception in music and dance.

## Figures and Tables

**Table 1:** The movement sequences presented in Experiment 1. Limb displacements were calculated for movement sequences in the middle tempo (IBI = 500 ms).

Movement type	Description	Limb displacement (cm)
Arms discrete	Arms performed *Toque de Palmas* on beats 1, 2, 3, 5, 6, and 7 of the 8-beat count. Legs stood still in basic position.	117.12

Arms continuous	The right arm performed *Braceo* during the 8-beat count. Legs in basic position.	239.71

Arms mixed	Two claps (*Toque de Palmas*) on beats 1 and 2, followed by both arms performing *Braceo* from beats 3 to 6 and then two more claps on beats 7 and 8. Legs in basic position.	415.00

Legs discrete	Legs performed *Zapateado *on beats 1, 2, 3, 5, 6, and 7. Arms remained still in basic position.	538.28

Legs continuous	The right leg was lifted up and down (beats 1 to 4), followed by a circular movement of the left leg (beats 5 to 8). Arms in basic position.	305.32

Legs mixed	The left foot tapped on beats 1 and 2, followed by a circular movement of the same leg during beats 3 to 6, and then two more taps on beats 7 and 8. Arms in basic position.	350.92

**Table 2:** The movement sequences presented in Experiment 2.

Movement type	Description
Arms discrete + legs discrete	Left foot tapped on beat 1 followed by *Toque de Palmas* of the arms on beats 2, 3, and 4; right foot tapped on beat 5 followed by *Toque de *Palmas of the arms on beats 6, 7, and 8.

Arms discrete + legs continuous	The hands clapped on beats 1, 2, and 3 and 5, 6, and 7, with the arms moving from the right to the left diagonal. In parallel, the legs made a continuous movement as in Experiment 1.

Arms continuous + legs discrete	The left arm performed *Braceo* while the legs performed *Marcaje*, both covering 8 beats.

Arms continuous + legs continuous	The right arm performed *Braceo* from beats 1 to 4 while the right foot carried out a circular movement, and then the pattern was repeated with the left arm and the left foot from beats 5 to 8.

## References

[1] Patel A. D. (2014). Can nonlinguistic musical training change the way the brain processes speech? The expanded OPERA hypothesis. *Hearing Research*.

[2] Allman M. J., Teki S., Griffiths T. D., Meck W. H. (2014). Properties of the internal clock: first- and second-order principles of subjective time. *Annual Review of Psychology*.

[3] Grahn J. A. (2012). Neural mechanisms of rhythm perception: current findings and future perspectives. *Topics in Cognitive Science*.

[4] Rathcke T. V., Smith R. H. (2015). Speech timing and linguistic rhythm: on the acoustic bases of rhythm typologies. *Journal of the Acoustical Society of America*.

[5] MacDougall H. G., Moore S. T. (2005). Marching to the beat of the same drummer: the spontaneous tempo of human locomotion. *Journal of Applied Physiology*.

[6] Burger B., Thompson M. R., Luck G., Saarikallio S. H., Toiviainen P. (2014). Hunting for the beat in the body: on period and phase locking in music-induced movement. *Frontiers in Human Neuroscience*.

[7] Merchant H., Pérez O., Zarco W., Gámez J. (2013). Interval tuning in the primate medial premotor cortex as a general timing mechanism. *The Journal of Neuroscience*.

[8] Ivry R. B., Hazeltine R. E. (1995). Perception and production of temporal intervals across a range of durations: evidence for a common timing mechanism. *Journal of Experimental Psychology: Human Perception and Performance*.

[9] Schubotz R. I., Friederici A. D., von Cramon D. Y. (2000). Time perception and motor timing: a common cortical and subcortical basis revealed by fMRI. *NeuroImage*.

[10] Teki S., Grube M., Griffiths T. D. (2012). A unified model of time perception accounts for duration-based and beat-based timing mechanisms. *Frontiers in Integrative Neuroscience*.

[11] Su Y.-H., Pöppel E. (2012). Body movement enhances the extraction of temporal structures in auditory sequences. *Psychological Research*.

[12] Manning F., Schutz M. (2013). ‘Moving to the beat’ improves timing perception. *Psychonomic Bulletin and Review*.

[13] Grahn J. A., Brett M. (2007). Rhythm and beat perception in motor areas of the brain. *Journal of Cognitive Neuroscience*.

[14] Grube M., Griffiths T. D. (2009). Metricality-enhanced temporal encoding and the subjective perception of rhythmic sequences. *Cortex*.

[15] Hove M. J., Fairhurst M. T., Kotz S. A., Keller P. E. (2013). Synchronizing with auditory and visual rhythms: an fMRI assessment of modality differences and modality appropriateness. *NeuroImage*.

[16] Grahn J. A., Henry M. J., McAuley J. D. (2011). FMRI investigation of cross-modal interactions in beat perception: audition primes vision, but not vice versa. *NeuroImage*.

[17] Grahn J. A. (2012). See what I hear? Beat perception in auditory and visual rhythms. *Experimental Brain Research*.

[18] Su Y.-H. (2014). Peak velocity as a cue in audiovisual synchrony perception of rhythmic stimuli. *Cognition*.

[19] Su Y.-H. (2014). Visual enhancement of auditory beat perception across auditory interference levels. *Brain and Cognition*.

[20] Lacquaniti F., Carrozzo M., D'Avella A., La Scaleia B., Moscatelli A., Zago M. (2014). How long did it last? You would better ask a human. *Frontiers in Neurorobotics*.

[21] Carrozzo M., Moscatelli A., Lacquaniti F. (2010). Tempo Rubato?: animacy speeds up time in the brain. *PLoS ONE*.

[22] Pozzo T., Papaxanthis C., Petit J. L., Schweighofer N., Stucchi N. (2006). Kinematic features of movement tunes perception and action coupling. *Behavioural Brain Research*.

[23] Stadler W., Springer A., Parkinson J., Prinz W. (2012). Movement kinematics affect action prediction: comparing human to non-human point-light actions. *Psychological Research*.

[24] Jeannerod M. (2001). Neural simulation of action: a unifying mechanism for motor cognition. *NeuroImage*.

[25] Wittmann M., van Wassenhove V. (2009). The experience of time: neural mechanisms and the interplay of emotion, cognition and embodiment. *Philosophical Transactions of the Royal Society B: Biological Sciences*.

[26] Naveda L., Leman M. (2010). The spatiotemporal representation of dance and music gestures using topological gesture analysis (TGA). *Music Perception*.

[27] Kirsch L. P., Cross E. S. (2015). Additive routes to action learning: layering experience shapes engagement of the action observation network. *Cerebral Cortex*.

[28] Sgouramani H., Vatakis A. (2014). ‘Flash’ dance: how speed modulates perceived duration in dancers and non-dancers. *Acta Psychologica*.

[29] Schaefer R. S. (2014). Images of time: temporal aspects of auditory and movement imagination. *Frontiers in Psychology*.

[30] Calvo-Merino B., Jola C., Glaser D. E., Haggard P. (2008). Towards a sensorimotor aesthetics of performing art. *Consciousness and Cognition*.

[31] Cross E. S., Kirsch L., Ticini L. F., Schütz-Bosbach S. (2011). The impact of aesthetic evaluation and physical ability on dance perception. *Frontiers in Human Neuroscience*.

[32] Brainard D. H. (1997). The psychophysics toolbox. *Spatial Vision*.

[33] Mioni G., Stablum F., McClintock S. M., Grondin S. (2014). Different methods for reproducing time, different results. *Attention, Perception, and Psychophysics*.

[34] Brown S. W. (1997). Attentional resources in timing: interference effects in concurrent temporal and nontemporal working memory tasks. *Perception and Psychophysics*.

[35] Jones M. R., Mcauley J. D. (2005). Time judgments in global temporal contexts. *Perception and Psychophysics*.

[36] McAuley J. D., Miller N. S. (2007). Picking up the pace: effects of global temporal context on sensitivity to the tempo of auditory sequences. *Perception and Psychophysics*.

[37] Gamache P.-L., Grondin S. (2010). Sensory-specific clock components and memory mechanisms: investigation with parallel timing. *European Journal of Neuroscience*.

[38] Smith D., Wakefield C. (2013). A timely review of a key aspect of motor imagery: a commentary on Guillot et al. (2012). *Frontiers in Human Neuroscience*.

[39] Guttman S. E., Gilroy L. A., Blake R. (2005). Hearing what the eyes see: auditory encoding of visual temporal sequences. *Psychological Science*.

[40] Povel D. J., Essens P. (1985). Perception of temporal patterns. *Music Perception*.

[41] Saygin A. P., Stadler W. (2012). The role of appearance and motion in action prediction. *Psychological Research*.

[42] Filippopoulos P. C., Hallworth P., Lee S., Wearden J. H. (2013). Interference between auditory and visual duration judgements suggests a common code for time. *Psychological Research*.

[43] Studenka B. E., Zelaznik H. N. (2011). Synchronization in repetitive smooth movement requires perceptible events. *Acta Psychologica*.

[44] Delignieres D., Torre K. (2011). Event-based and emergent timing: dichotomy or continuum? a reply to repp and steinman (2010). *Journal of Motor Behavior*.

[45] Grondin S., Killeen P. R. (2009). Tracking time with song and count: different Weber functions for musicians and nonmusicians. *Attention, Perception, and Psychophysics*.

[46] Rossit S., McAdam T., Mclean D. A., Goodale M. A., Culham J. C. (2013). FMRI reveals a lower visual field preference for hand actions in human superior parieto-occipital cortex (SPOC) and precuneus. *Cortex*.

[47] Kraft A., Sommer W. H., Schmidt S., Brandt S. A. (2011). Dynamic upper and lower visual field preferences within the human dorsal frontoparietal attention network. *Human Brain Mapping*.

[48] Iversen J. R., Patel A. D., Nicodemus B., Emmorey K. (2015). Synchronization to auditory and visual rhythms in hearing and deaf individuals. *Cognition*.

[49] Su Y.-H., Jonikaitis D. (2011). Hearing the speed: visual motion biases the perception of auditory tempo. *Experimental Brain Research*.

